# Author Reply: ‘Is lung function in a race against time?’

**DOI:** 10.1113/EP093720

**Published:** 2026-02-19

**Authors:** Bredan Cooper, Sanja Stanojevic

**Affiliations:** ^1^ Lung Function & Sleep, Queen Elizabeth Hospital University Hospitals Birmingham NHSFT Birmingham UK; ^2^ Department of Community Health and Epidemiology Dalhousie University Halifax Nova Scotia Canada

**Keywords:** equity, reference equations, spirometry

1

In his recent Letter, Mr Wallbanks makes several excellent points and further expands on our original discussion about the use of race and ethnic specific reference equations for lung function (Wallbanks, 2026). We completely agree that the factors that contribute to the growth and development of the lungs across the life course are complex and that shared genetic and environmental factors may explain some of the differences in lung function between populations. The respiratory community urgently needs to co‐ordinate to conduct high‐quality studies in diverse and globally representative populations to identify modifiable factors that can help to improve lung health for all.

The point Mr Wallbanks makes about clinical decision‐making is valid. The recognition of the limitations of *all* reference equations and how their use affects clinical decisions requires the community to re‐evaluate current threshold‐based decisions and ensure that guidelines are based on valid data and rigorous methods. It is worth noting that multiple publications have demonstrated that the GLI race‐neutral approaches more closely align with symptom burden, pulmonary exacerbations in COPD, radiological abnormalities and mortality across multiple ethnic groups (Baugh et al., 2023; Baugh et al., 2022; Brems et al., 2023; Brems et al., 2024; Elmaleh‐Sachs et al., 2022; McCormack et al., 2022; Rajaram et al., 2025; Regan et al., 2024; Sheshadri et al., 2024). The limitations raised regarding the GLI race‐neutral equations highlight that the respiratory community has done a poor job including people of diverse backgrounds in research and in the population‐based studies required to generate reference equations. Further, all reference equations have been derived using an outdated concept of health which ignores myriad of factors that influence lung health across the life course. As a community we must do better.

We agree with Mr Wallbanks, if we are to truly address inequities we must start at the beginning and question why and how we use pulmonary function test results.  A truly equitable approach is much more complex than the decision to use GLI global or race‐specific equations. We must question our assumption about what ‘healthy’ is, and whether current guidelines accurately and equitably identify lung conditions.

## AUTHOR CONTRIBUTIONS

All authors have approved the final version of the manuscript and agree to be accountable for all aspects of the work in ensuring that questions related to the accuracy or integrity of any part of the work are appropriately investigated and resolved. All persons designated as authors qualify for authorship, and all those who qualify for authorship are listed

## CONFLICT OF INTEREST

None declared.

## FUNDING INFORMATION

None.
